# Evaluation of Serum Endocan and Testosterone Levels in Male Patients with Prediabetes: A Cross-Sectional Clinical Study

**DOI:** 10.3390/jcm15041597

**Published:** 2026-02-19

**Authors:** Onur Selcuk Yigit, Mehmet Fatih Uzanulu, Selin Genc, Ibrahim Sahin

**Affiliations:** Department of Internal Medicine, Inonu University Faculty of Medicine, 44280 Malatya, Turkey; mehmet.uzaulu@ozal.edu.tr (M.F.U.); selin.genc@saglik.gov.tr (S.G.); ibrahimsahin@memorial.com.tr (I.S.)

**Keywords:** endocan, testosterone, prediabetes, insulin resistance, endothelial dysfunction, type 2 diabetes mellitus

## Abstract

**Background/Objectives:** This study aimed to investigate the relationship between serum endocan and total testosterone levels in male patients with prediabetes. **Materials and Methods:** This cross-sectional observational study included 37 men with prediabetes and 37 healthy male controls. In addition to routine laboratory tests, blood samples were collected to measure serum endocan levels using the enzyme-linked immunosorbent assay (ELISA), and total testosterone levels were analyzed using a chemiluminescence method. **Results:** Age did not differ significantly between the groups (*p* > 0.05). Body mass index (BMI), fasting plasma glucose (FPG), postprandial plasma glucose (PPG), insulin, HbA1c, and serum endocan levels were significantly higher in the prediabetes group (BMI: *p* = 0.003; FPG: *p* < 0.001; PPG: *p* = 0.019; insulin: *p* = 0.007; HbA1c: *p* < 0.001; endocan: *p* = 0.012). No significant difference was observed in testosterone levels between the groups (*p* = 0.228). **Conclusions:** Elevated serum endocan levels in individuals with prediabetes may reflect early endothelial dysfunction associated with glycemic dysregulation. These findings suggest that endocan may serve as an exploratory biomarker of early vascular alterations in prediabetes. However, further large-scale and prospective studies are warranted to clarify its clinical relevance and potential role in risk stratification and the prediction of microvascular complications.

## 1. Introduction

Prediabetes is an intermediate metabolic state characterized by an increased risk of developing diabetes, in which fasting plasma glucose, postprandial glucose, or HbA1c levels fall between normal values and diagnostic thresholds for diabetes [1]. The prevalence of prediabetes has been steadily rising and poses a significant public health concern [2,3]. As an early stage of type 2 diabetes, approximately 70% of individuals with prediabetes eventually progress to diabetes. Previous studies have demonstrated that diabetes-related complications may begin during the prediabetic stage, and that these complications can be reduced by 40–58% through lifestyle interventions alone [4,5,6]. Therefore, the prediabetic period represents a critical window for intervention, making the accurate identification of metabolic and vascular alterations highly important for preventing diabetes and its associated complications.

In individuals with prediabetes, increased oxidative stress, inflammation, insulin resistance, and glucose toxicity may contribute to the development of diabetic microvascular complications, albeit at a lower frequency compared to overt diabetes [7]. From a clinical perspective, the investigation of biomarkers reflecting endothelial function may play a potential role in the early diagnosis and risk stratification of individuals with prediabetes.

Endocan (endothelial cell-specific molecule-1; ESM-1) is a 50-kDa proteoglycan synthesized by vascular endothelial cells and is associated with inflammation and angiogenesis [8,9,10]. Studies in patients with hypertension, sepsis, acute respiratory distress syndrome (ARDS), malignancies, atherosclerosis, and diabetes mellitus have reported elevated serum endocan levels compared with healthy controls [11,12]. Mechanistically, endocan increases the expression of adhesion molecules such as ICAM-1 and VCAM-1, which facilitate leukocyte adhesion and transendothelial migration, while suppression of endocan expression reduces these adhesion molecules and inhibits monocyte–endothelial interactions [13,14]. Therefore, endocan may serve as a novel biomarker for endothelial dysfunction and disease severity in disorders with vascular endothelial involvement.

Hormonal alterations also contribute to the development of prediabetes. Several studies have demonstrated associations between low testosterone levels and endothelial dysfunction, coronary artery disease, metabolic syndrome, and diabetes mellitus [15,16,17,18]. Testosterone influences glucose metabolism, insulin sensitivity, and abdominal fat distribution, and low testosterone has been identified as a predictive factor for coronary artery disease [19]. Testosterone may additionally exert protective effects by increasing fibrinolytic activity and reducing fibrinogen levels [15]. Although low testosterone levels are linked to type 2 diabetes and metabolic syndrome, the role of testosterone during the prediabetic stage remains incompletely understood [16,17,18].

Beyond glycemic abnormalities, prediabetes is increasingly recognized as a condition associated with early endothelial and microvascular alterations that may precede overt diabetes [4,6]. Identifying biomarkers that reflect subclinical endothelial dysfunction during this stage may provide opportunities for early risk stratification and timely preventive interventions before irreversible microvascular damage occurs.

Testosterone plays an important role in glucose metabolism, insulin sensitivity, and body fat distribution, and accumulating evidence indicates that low serum testosterone levels are associated with endothelial dysfunction, coronary artery disease, metabolic syndrome, and the development of diabetes mellitus [15,16,17,18]. However, the relationship between testosterone and endothelial biomarkers during the prediabetic stage has not been clearly elucidated. To minimize hormonal heterogeneity and reduce potential confounding related to sex-specific differences in sex hormone regulation, the present study focused exclusively on male participants. Accordingly, investigating the relationship between endocan, a marker of endothelial dysfunction, and testosterone levels in men with prediabetes may provide clinically relevant insights into early vascular alterations associated with dysglycemia.

The aim of this study was to investigate the relationship between serum endocan and testosterone levels in male individuals with prediabetes. Identifying early vascular alterations during the prediabetic period and determining whether endocan may serve as a biomarker for prediabetes could support the development of early screening strategies in clinical practice.

## 2. Materials and Methods

### 2.1. Study Design

This study was designed as a cross-sectional observational study. All clinical, biochemical, and hormonal measurements were obtained at a single time point.

### 2.2. Study Population

Ethics committee approval was obtained at the beginning of the study, and written informed consent was obtained from all participants. All procedures were conducted in accordance with the principles of the Declaration of Helsinki.

The study was conducted between 1 January 2022 and 1 August 2022, in male individuals aged 20–65 years who presented to the Endocrinology and Diabetes Outpatient Clinics of the Department of Internal Medicine at Inonu University Faculty of Medicine. The study included 37 patients diagnosed with prediabetes and 37 healthy individuals with no history of chronic disease, malignancy, regular medication use, smoking, or alcohol consumption, and no known psychiatric disorders.

The control group consisted of individuals who presented to the outpatient clinics for routine check-ups and were selected on a voluntary basis. Prediabetes was defined according to the American Diabetes Association (ADA) criteria as the presence of at least one of the following: fasting plasma glucose (FPG) levels between 100 and 125 mg/dL, postprandial plasma glucose (PPG) levels between 140 and 199 mg/dL, or HbA1c values between 5.7% and 6.4% [20]. Oral glucose tolerance testing (OGTT) was not routinely performed.

Participants using medications known to affect glucose metabolism, insulin sensitivity, inflammation or hormonal status were excluded. These included systemic corticosteroids, antidepressants, antipsychotics, anti-androgenic agents, and any form of testosterone or hormonal replacement therapy. In addition, individuals reporting regular use of over-the-counter supplements with potential metabolic or hormonal effects were excluded. Medication use and hormonal history were assessed in detail based on medical history obtained at enrollment, and participants with a history of hypogonadism or recent hormonal treatment were not included in the study.

### 2.3. Sample Collection and Laboratory Analysis

Venous blood samples were collected from all participants in the morning between 09:00 and 10:00 a.m. after an overnight fast during routine evaluations. After allowing the samples to clot for 30 min, the venous blood samples were centrifuged at 2000× *g* for 10 min, and the obtained sera were stored at −80 °C until analysis.

Serum endocan (ESM-1) levels were measured using a commercially available sandwich enzyme-linked immunosorbent assay (ELISA) kit (Human ESM1/Endothelial Cell Specific Molecule-1 ELISA Kit; Elabscience^®^, Houston, TX, USA; Cat. No: E-EL-H1557), in the Research Laboratory of the Department of Biochemistry at İnönü University Faculty of Medicine, in accordance with the manufacturer’s instructions. Absorbance was measured at 450 nm using a BioTek SYNERGY H1 microplate reader (BioTek Instruments, Winooski, VT, USA).

According to the manufacturer’s performance data, intra-assay precision was evaluated by measuring three samples with low, medium, and high ESM-1 concentrations 20 times within the same plate, yielding coefficients of variation (CVs) of 6.36%, 5.69%, and 3.07%, respectively. Inter-assay precision was assessed by measuring the same three concentration levels on three different plates (20 replicates per plate), with corresponding CVs of 6.09%, 5.98%, and 5.06%, indicating acceptable assay reproducibility.

Serum testosterone levels were analyzed using the chemiluminescence method on a Beckman Coulter UniCel DxI 800 analyzer (Beckman Coulter, Brea, CA, USA). Metabolic parameters, including fasting plasma glucose (FPG), postprandial plasma glucose (PPG), HbA1c, lipid profile, insulin levels, and homeostasis model assessment of insulin resistance (HOMA-IR), were obtained from routine laboratory evaluations. Height and weight were recorded, and body mass index (BMI) was calculated as kg/m^2^.

### 2.4. Statistical Analysis

Statistical analyses were performed using IBM SPSS Statistics software version 22.0. The normality of continuous variables was assessed using the Shapiro–Wilk test. Variables with a normal distribution were expressed as mean ± standard deviation, whereas non-normally distributed variables were presented as median (minimum–maximum) values. Comparisons between groups were performed using Student’s *t*-test or the Mann–Whitney U test, as appropriate.

Receiver operating characteristic (ROC) curve analysis was conducted to determine the diagnostic value of endocan levels for prediabetes, and area under the curve (AUC) values were calculated. Logistic regression analysis was used to evaluate independent variables associated with the development of prediabetes. Given the limited number of outcome events, multivariable logistic regression was not performed to avoid model overfitting. A *p* value < 0.05 was considered statistically significant.

The optimal cut-off value for serum endocan levels was determined using the Youden index, which maximizes the sum of sensitivity and specificity. Confidence intervals for the area under the curve (AUC) were calculated as part of the ROC analysis. No internal or external validation was performed, and the identified cut-off value should therefore be interpreted cautiously.

Sample size estimation was performed prior to the initiation of the study based on serum endocan levels, which were defined as the primary outcome of the study. Using an assumed effect size of 0.72 derived from previously published data by Klisić et al. in patients with type 2 diabetes mellitus [21], a two-sided alpha level of 0.05, and a desired statistical power (1 − β) of 0.80, the minimum required minimum sample size was calculated as 32 participants per group, resulting in a total sample size of 64 subjects. With a group size of 32 participants, the achieved power was calculated as 0.806. Sample size and power calculations were performed using the web-based Sample Size and Power Analysis application (WSSPAS) developed by Inonu University [22].

## 3. Results

A total of 74 male participants were included in the study, with 37 individuals in the prediabetes group and 37 in the control group. The overall mean age of the participants was 41.31 ± 6.60 years, and no significant difference in age was observed between the groups (*p* > 0.05). Body mass index (BMI) was significantly higher in the prediabetes group compared with the control group (*p* < 0.05).

Fasting plasma glucose (FPG), HbA1c, insulin, and HOMA-IR levels were significantly higher in the prediabetes group compared with the control group (all *p* < 0.001). Although higher triglyceride levels and lower HDL cholesterol levels were observed in the prediabetes group, these differences were not statistically significant (Table 1).

**Figure 1:**
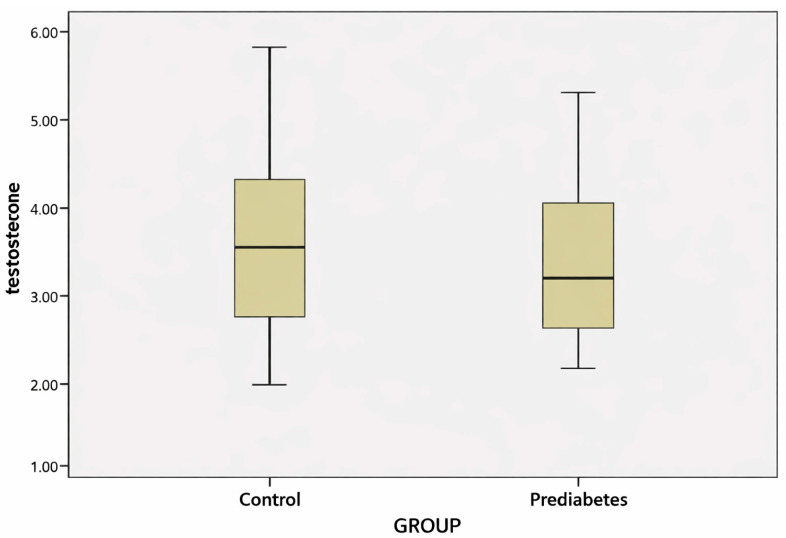
Distribution of serum testosterone levels in the prediabetes and control groups (median–IQR).

Serum endocan levels were significantly higher in the prediabetes group compared with the control group (588.59 ± 475.49 ng/L vs. 381.04 ± 257.57 ng/L; *p* = 0.012) (Table 1). In contrast, no significant difference was observed between the two groups in terms of testosterone levels (*p* = 0.228) (Figure 1).

**Figure 2:**
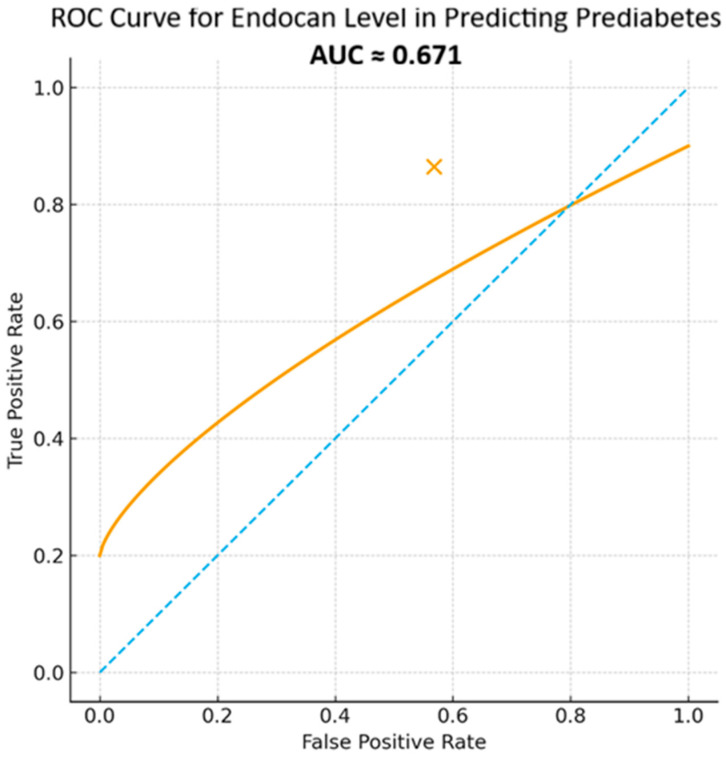
ROC curve for endocan. The blue dotted line represents the no-discrimination reference line (AUC = 0.5).

Receiver operating characteristic (ROC) curve analysis, performed to evaluate the predictive performance of endocan levels for prediabetes, revealed an area under the curve (AUC) of 0.671, which was statistically significant (AUC = 0.671; 95% CI: 0.548–0.793; *p* = 0.012) (Figure 2).

Different cut-off values for endocan were evaluated, and the optimal cut-off value was calculated as 227.99 ng/L in the present study. Values above this threshold may be interpreted in favor of prediabetes. At the determined cut-off value, sensitivity was 86.5% and specificity was 43.2%.

In the binary logistic regression analysis performed to evaluate prediabetes risk, the model was found to be statistically significant (Omnibus test *p* = 0.031). Only the endocan category was included in the analysis as an independent variable. For endocan, individuals with values below the proposed cut-off (<227.99 ng/L) were defined as the reference group, whereas those with values above this threshold were classified as the risk group.

**Figure 3:**
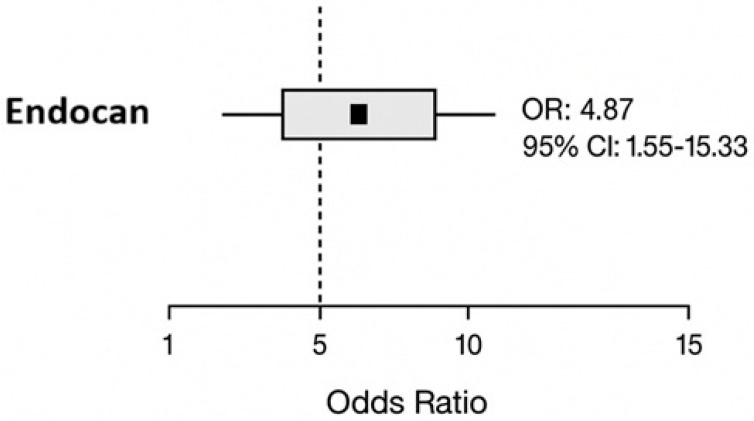
Forest plot of the logistic regression analysis demonstrating the association between endocan levels and prediabetes.

The model demonstrated a moderate explanatory power for prediabetes (Nagelkerke R^2^ = 0.142). The analysis showed that endocan levels were independently associated with the development of prediabetes. Individuals with endocan levels ≥ 227.99 ng/L had a significantly increased risk of prediabetes (OR: 4.876; 95% CI: 1.551–15.325, *p* = 0.007) (Figure 3).

## 4. Discussion

In this study, serum endocan and testosterone levels were evaluated in male individuals with prediabetes, and metabolic alterations associated with prediabetes were examined. The findings demonstrated that serum endocan levels were significantly higher in the prediabetes group compared with healthy controls. This suggests that endothelial dysfunction may begin during the prediabetic stage and that endocan may represent a relevant biomarker reflecting early vascular injury. Since prediabetes is considered an early stage of type 2 diabetes and cardiometabolic disease, identifying biological markers that capture endothelial and microvascular alterations during this period is of considerable clinical importance.

Several studies have reported elevated endocan levels in individuals with type 2 diabetes mellitus (T2D) [23,24,25]. However, studies comparing endocan levels between individuals with prediabetes and healthy controls have yielded inconsistent results. Klisić et al. reported that median endocan levels were slightly higher in prediabetic individuals than in healthy subjects, although the difference was not statistically significant [26]. Conversely, Arman et al. found significantly lower endocan levels in prediabetic individuals compared with normoglycemic controls [23]. In line with these variations, Cassano et al. demonstrated that endocan levels were significantly higher in individuals with normoglycemia who exhibited 1-h post-oral glucose tolerance test (OGTT) plasma glucose ≥ 155 mg/dL, suggesting that glucose dysregulation may precede overt hyperglycemia [27].

Although there is general agreement that endocan levels are markedly elevated in T2D, alterations during the prediabetic stage may differ depending on study methodology, metabolic characteristics of the population, and diagnostic criteria used to define prediabetes. The findings of the present study support the hypothesis that endocan may serve as an indicator of endothelial stress during prediabetes.

A recent systematic review and meta-analysis by Khalaji et al. reported higher serum endocan levels in individuals with diabetes compared with healthy controls [28]. However, the authors also emphasized substantial heterogeneity among the included studies, likely reflecting differences in study populations, disease duration, metabolic status, and analytical methods. Although the pooled effect estimates did not cross the null value, the relatively wide confidence intervals indicate variability in the magnitude of the observed association. Taken together, these findings suggest that elevated endocan levels may be associated with diabetes-related vascular alterations, but they should be interpreted with caution. Accordingly, extrapolation of these results to earlier stages of dysglycemia, such as prediabetes, requires careful consideration. Within this context, the present study provides complementary evidence by specifically examining endocan levels during the prediabetic stage.

The positive association observed between endocan and HbA1c levels in our study is also clinically relevant. HbA1c reflects cumulative glycemic exposure and is linked to vascular injury related to chronic hyperglycemia. The correlation between HbA1c and endocan suggests that endocan may increase under metabolic stress and reflect vascular consequences of glycemic deterioration. This supports the notion that prediabetes is not merely a biochemical abnormality in glucose regulation but also encompasses early endothelial impairment.

When evaluating the diagnostic performance of endocan for prediabetes using ROC analysis, the AUC value was 0.671, with a cut-off value of 227.99 ng/L. Although endocan demonstrated limited specificity as a diagnostic marker, its relatively high sensitivity may be useful for identifying subclinical metabolic alterations. Logistic regression analysis showed that higher serum endocan levels were associated with an increased likelihood of prediabetes. However, given the moderate discriminatory performance observed in ROC analysis, endocan should not be considered a standalone diagnostic tool. Furthermore, due to the relatively small sample size and the limited number of outcome events, inclusion of multiple covariates in a multivariable logistic regression model was not performed, as this could violate recommended events-per-variable assumptions and increase the risk of model overfitting and unstable estimates. Therefore, the observed associations should be interpreted with caution, and further studies with larger cohorts are warranted to clarify the role of endocan in early risk stratification.

In this study, testosterone levels did not differ significantly between the prediabetes and control groups. Although multiple studies have demonstrated associations between low testosterone, metabolic syndrome, and T2D, findings related to the prediabetic stage remain inconsistent. Some studies have reported lower testosterone levels in individuals with prediabetes, whereas others have found no significant differences [29,30]. The absence of a significant difference in testosterone levels in our study suggests that hormonal changes may not be prominent during the early stages of metabolic disruption, or that the sample size may have been insufficient to detect such differences. Additionally, the lack of an independent association between testosterone and prediabetes in regression analysis suggests that endothelial biomarkers may play a more dominant role during early glycemic impairment.

As expected, metabolic parameters including fasting plasma glucose, HbA1c, insulin, and HOMA-IR were significantly higher in the prediabetes group. In addition, the tendency toward higher triglyceride levels and lower HDL cholesterol levels is consistent with well-established associations between prediabetes and atherogenic dyslipidemia, further highlighting the close relationship between prediabetes and metabolic syndrome.

The strengths of this study include the use of a reliable laboratory method (ELISA) for biomarker quantification, an age-matched control design, and a specific focus on the prediabetic stage. However, several limitations should be acknowledged. Given the cross-sectional design of the study, causal relationships between endocan levels and metabolic parameters cannot be established. Additionally, due to the limited sample size, comprehensive multivariable adjustment in regression analyses was not feasible without risking model overfitting. In addition, the relatively small sample size and the inclusion of only male participants limit the generalizability of the findings to broader populations. Prospective studies with larger and more diverse cohorts are therefore warranted to more comprehensively evaluate the role of endocan in prediabetes and its potential as a screening biomarker for early microvascular risk.

Furthermore, physical activity levels were not quantitatively assessed, which may have acted as a residual confounder influencing metabolic parameters, endothelial function, and hormonal status. Finally, because the sample size calculation was based on serum endocan levels as the primary outcome, the study may not have been sufficiently powered to detect small-to-moderate differences in testosterone levels; therefore, testosterone-related findings should be interpreted with caution.

## 5. Conclusions

This study demonstrated that serum endocan levels are significantly elevated in male individuals with prediabetes and that endocan is independently associated with the presence of prediabetes. The positive correlation between HbA1c and endocan further supports the concept that endothelial dysfunction begins during the early stages of glycemic deterioration. These findings suggest that microvascular injury may arise at a subclinical level during the prediabetic period, and that endocan may serve as a potential early warning biomarker in this context. Conversely, no significant association was observed between testosterone levels and prediabetes. Larger, prospective studies with longer follow-up periods are needed to more clearly define the diagnostic and prognostic value of endocan in the clinical management of prediabetes. However, given the moderate discriminatory performance observed in ROC analysis and the cross-sectional design of the study, these findings should be interpreted with caution, and future prospective studies are required to validate the potential role of endocan in early risk stratification.

## Figures and Tables

**Table 1:** Comparison of demographic and biochemical characteristics between the prediabetes and control groups.

	GROUP				
	Prediabetes		Control		
	Mean ± Standard Deviation	Median	Mean ± Standard Deviation	Median	*p* Value
**Age (years)**	43.19 ± 5.89	43.00	39.43 ± 6.81	40.00	0.13
**BMI (kg/m^2^)**	29.33 ± 4.16	28.71	26.59 ± 3.45	26.51	**0.003**
**FPG (mg/dL)**	98.62 ± 13.02	95.00	86.92 ± 6.22	87.00	**<0.001**
**PPG (mg/dL)**	115.70 ± 30.36	106.00	98.35 ± 14.49	99.00	**0.019**
**TG (mg/dL)**	155.24 ± 78.29	143.00	165.05 ± 93.70	149.00	0.871
**Cholesterol (mg/dL)**	193.24 ± 30.90	195.00	191.62 ± 42.21	188.00	0.851
**HDL (mg/dL)**	43.56 ± 9.85	40.00	44.35 ± 9.99	42.00	0.506
**LDL (mg/dL)**	119.86 ± 24.72	111.00	114.13 ± 33.62	118.00	0.486
**VLDL (mg/dL)**	30.16 ± 15.37	27.00	33.06 ± 19.13	30.60	0.705
**Insulin (mU/L)**	20.15 ± 30.05	12.50	9.42 ± 5.03	7.80	**0.007**
**HbA1c (%)**	5.88 ± 0.17	5.80	5.34 ± 0.21	5.30	**<0.001**
**Creatinine (mg/dL)**	0.95 ± 0.10	0.93	0.96 ± 0.16	1.00	0.926
**AST (mU/L)**	23.03 ± 7.82	21.00	22.43 ± 5.14	22.00	0.672
**ALT (mU/L)**	31.65 ± 15.43	27.00	27.38 ± 11.13	26.00	0.176
**HOMA-IR**	3.96 ± 2.77	3.16	1.92 ± 1.19	1.69	**<0.001**
**Testosterone (ng/dL)**	3.26 ± 0.84	3.12	3.51 ± 0.96	3.45	0.228
**Endocan (ng/L)**	588.59 ± 475.49	442.14	381.04 ± 257.57	257.20	**0.012**

BMI: Body mass index; HOMA-IR: Homeostatic Model Assessment of Insulin Resistance; HDL: High-density lipoprotein; LDL: Low-density lipoprotein; AST: Aspartate aminotransferase; ALT: Alanine aminotransferase. HbA1c: Glycated Hemoglobin; TG: Triglycerides; VLDL: Very-low-density lipoprotein; Bold values indicate statistical significance (*p* < 0.05).

## Data Availability

The data presented in this study are available from the corresponding author upon reasonable request.

## Abbreviations

The following abbreviations are used in this manuscript:

ADA	American Diabetes Association
BMI	Body Mass Index
ELISA	Enzyme Linked Immunosorbent Assay
ESM 1	Endothelial Cell Specific Molecule 1
FPG	Fasting Plasma Glucose
HbA1c	Glycated Hemoglobin
HDL	High Density Lipoprotein
HOMA IR	Homeostatic Model Assessment of Insulin Resistance
LDL	Low Density Lipoprotein
PPG	Postprandial Plasma Glucose
ROC	Receiver Operating Characteristic
